# Immobilization of Lead and Zinc in Tailings Sand Using a Stabilizer Synthesized from Granite Sawdust for Mine Remediation

**DOI:** 10.3390/ma19010199

**Published:** 2026-01-05

**Authors:** Yanping Shi, Mengjia Liang, Man Xue, Zhi Li, Xianyu Yang, Chuyuan Ma, Longchen Duan, Jihua Cai

**Affiliations:** 1 College of Engineering, China University of Petroleum-Beijing at Karamay, Karamay 834000, China; 2 Faculty of Engineering, China University of Geosciences, Wuhan 430074, China

**Keywords:** granite sawdust, zeolite-based stabilizer, alkali fusion–hydrothermal method, heavy metal tailings waste

## Abstract

Improper disposal of granite sawdust from stone processing and heavy metal-containing tailings sand can pose severe threats to the environment and human health. Based on their physicochemical properties, granite sawdust was used to synthesize a zeolite-based stabilizer (GFAS) for immobilizing lead (Pb) and zinc (Zn) in tailings waste. The stabilizer was prepared through an alkali fusion–hydrothermal method, followed by phosphoric acid modification. Characterization by XRD, SEM-EDS, and BET revealed that GFAS possesses a Na-P1 zeolite structure (Na_6_Al_6_Si_10_O_32_) with a micro-mesoporous texture and a specific surface area of 35.00 m^2^/g, representing a 10-fold increase over raw sawdust. The cation exchange capacity (CEC) of GFAS reached 57.08 cmol^+^/kg, a 116-fold enhancement. The stabilization mechanism involved synergistic physical adsorption, chemical precipitation (e.g., Pb_3_(PO_4_)_2_, Zn(OH)_2_), and ion exchange. This study presents a sustainable “waste-treats-waste” strategy for effectively reducing the mobility of heavy metals in tailings waste, thereby contributing to the remediation of seepage from tailings pond foundations.

## 1. Introduction

Granite sawdust is a solid waste generated during stone extraction in granite mines and during granite slab processing (e.g., cutting, grinding, and polishing). It primarily consists of stone particles, powder, minor metal fragments, and water [1,2]. Globally, the quarrying and processing of dimension stone produce enormous amounts of waste, estimated to be around 175 million tons annually [3]. The waste residue from granite processing, predominantly fine-grained sawdust generated during stone-cutting, accounts for approximately 15~20% of the total quarried mass [4]. In many key production regions, this equates to the generation of hundreds of thousands of tonnes of granite fines every year [5,6]. Improper disposal of this waste has resulted in the loss of fertile farmland, soil erosion, increased respiratory diseases, and severe ecological degradation [7]. Consequently, developing effective treatment and utilization methods for this solid waste has become a pressing challenge within the industry.

**Figure 1:**
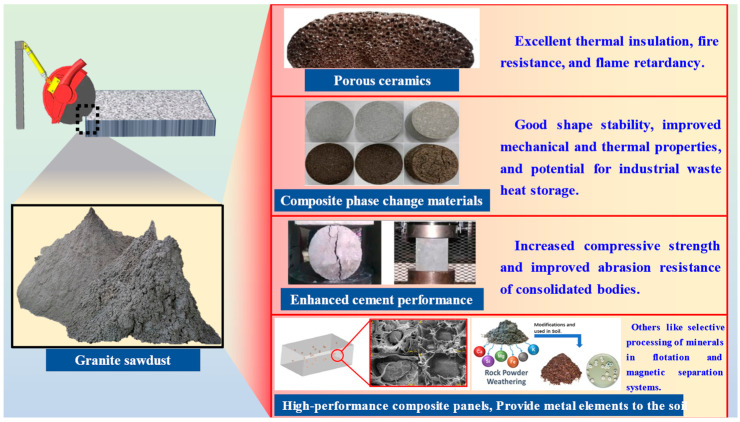
Application of granite sawdust in various fields.

The primary mineral constituents of granite sawdust are quartz, feldspar, and mica, with SiO_2_ and Al_2_O_3_ as the dominant oxides, accompanied by minor amounts of Na_2_O, CaO, and MgO [8,9]. Recent studies further characterize this material, confirming that the fine-grained rock waste from granite production is primarily composed of these silicate minerals, providing a strong elemental basis for its valorization [5]. The development of repurposing strategies for granite sawdust has gained significant attention. The current applications focus on its use as a secondary industrial raw material, in stone-like coatings, concrete, and material fabrication (e.g., ceramics, ceramic tiles, glass, geopolymers, and composite panels) (Figure 1) [10,11,12,13,14].

Furthermore, the granite waste is characterized by a high degree of liberation of mineral components, as well as fine grain size, which strongly enhances its potential as a raw material, especially in terms of the selective processing of minerals in flotation and magnetic separation systems. This potential extends to its possible use as a natural fertilizer or as a semi-finished product for producing purified quartz-feldspar flours for the ceramics industry [5,15]. At the same time, the high content of silicon and aluminum in granite sawdust provides an elemental foundation for synthesizing zeolite-based stabilizers. Zeolites, a class of microporous crystalline aluminosilicates, possess a suite of distinctive properties, including high cation exchange capacity, low density, substantial void volume, excellent thermal stability, and uniform molecular-sized channels, which enable a diverse range of industrial applications [16,17]. Their internal structure, featuring intricate pores and channels, confers unique adsorption and ion exchange capabilities. Owing to these characteristics, zeolites are widely employed as metal ion exchangers, catalysts, and adsorbents, playing critical roles in petroleum refining, petrochemicals, chemical manufacturing, agriculture, and wastewater treatment [18,19,20,21,22,23].

Heavy metal tailings ponds, as a storage facility for industrial waste during the mining of metal minerals, contain a large amount of heavy metals. Leakage from tailings ponds causes heavy metal pollution and biological toxicity in the surrounding environment [24,25,26,27,28,29,30]. Heavy metals undergo migration and transformation in the environment over time, during which they severely impact microbial diversity, soil quality, food security, and human health [31,32,33]. Consequently, there is an urgent need for effective stabilizers capable of adsorbing and immobilizing heavy metals in tailings waste, thereby reducing their environmental mobility and bioavailability to mitigate potential threats to ecosystems and human health.

In addressing heavy metal pollution, Bakalarz and Duchnowska analyzed the possibility of recovering copper from copper flotation tailings, demonstrating its potential as a secondary raw material source [6]. Usman et al. reported that an application rate of 1:50 (clay-to-soil ratio) resulted in the adsorption of 70% of heavy metal species [34]. Zhang et al. observed an effective clay dosage of 8%, while Jin et al. recorded effective doses of 2.5% or less [35,36]. Wang et al. combined sodium alginate with attapulgite, achieving maximum adsorption capacities of 119 mg/g for Cd^2+^ and 160 mg/g for Cu^2+^ [37]. Furthermore, Shi et al. effectively inhibited the migration of lead and zinc in tailings waste using composite-modified clay minerals [38].

However, as a silicon- and aluminum-rich solid waste derived from natural minerals, granite sawdust has rarely been investigated for synthesizing heavy metal stabilizers, and there has been limited research into its synthesis methods and pathways. Furthermore, the adsorption performance of the synthesized product for heavy metals in tailings waste lacks systematic research. Therefore, developing a stabilizer based on granite sawdust that exhibits high immobilization efficacy for heavy metals in tailings waste is of significant importance. This approach would not only expand the technical methods for the secondary utilization of granite sawdust but also achieve effective environmental remediation through a “waste-treats-waste” strategy.

This study first analyzed the physicochemical properties of heavy metal-containing tailings waste and granite sawdust. Subsequently, a heavy metal stabilizer was synthesized from granite sawdust using an alkali fusion–hydrothermal method. The adsorption capacity of the synthesized product for heavy metals in surface tailings waste from the Shenxiandong Lead-Zinc Tailing Reservoir in Guizhou Province was evaluated through leaching experiments. The stabilizer was further modified via acid treatment and phosphate group functionalization to develop an adsorbent with enhanced adsorption capability for lead and zinc in tailings waste, thereby improving the capacity of clay minerals to immobilize these heavy metals. Finally, the structure and underlying mechanisms were investigated using SEM, BET, CEC, and other analytical techniques.

## 2. Materials and Methods

### 2.1. Experimental Materials

Hydrochloric acid (37%, p.a. grade, HCl), phosphoric acid (85%, p.a. grade, H_3_PO_4_), and sodium hydroxide (≥98%, p.a. grade, NaOH) were procured from Jiangsu Taicang Hushi Reagent Co., Ltd. in Suzhou, China. Hexaamminecobalt(III) chloride (≥99%, p.a. grade, [Co(NH_3_)_6_]Cl_3_) was supplied by Bidepharm Co., Ltd., Shanghai, China. Gongyi Borun Refractory Co., Ltd., Zhengzhou, China was the source of the fly ash material. The tailing sand for this investigation was gathered from the surface layer of the Shenxiandong lead-zinc tailings pond. The site is geographically situated in Jianshui County, Honghe Hani and Yi Autonomous Prefecture, Yunnan Province (102°52′12″ E, 23°33′53″ N). The granite sawdust utilized was acquired from a stone slab processing facility located in Wuhan City, Hubei Province, China.

### 2.2. Experimental Methods

#### 2.2.1. Leaching Experiment of Tailings Waste

A 0.1 mol/L acetic acid solution, exhibiting a pH of 2.88 ± 0.05, served as the primary leaching medium. The heavy metal tailing samples were mixed with the leaching fluid at a solid-to-liquid mass ratio of 1:20. Subsequently, the resulting suspension was subjected to agitation for 24 h. Following a 20 min period of high-speed centrifugation, the clear supernatant was collected and passed through a 0.45 μm pore size microporous membrane for filtration [39]. The concentrations of the heavy metals Pb and Zn in the final filtrate were quantified utilizing PerkinElmer NexION 300X Inductively Coupled Plasma Mass Spectrometry (ICP-MS, Worcester, MA, USA). Given the dynamic nature of Pb and Zn elution from the tested tailings waste, the overall quantity of these two elements within the leaching solution was not measured. Reference samples were re-analyzed in every experimental run to serve as an internal control, thereby upholding the quality of the data and reducing potential experimental errors.

#### 2.2.2. Physical and Chemical Analysis of Tailings Waste

An X’Pert PRO DY2198 X-ray diffractometer (XRD, Almelo, The Netherlands) was employed to ascertain the mineral phases present in the tailings waste, while the leached quantities of Pb and Zn were obtained from the leaching test results. Sieving analysis was conducted to determine the particle size distribution of tailings waste collected from three distinct sampling locations within the specified area.

Specifically, a 500 g tailing sample was sequentially passed through a stack of sieves with the following mesh sizes (in ascending order): 2000, 850, 425, 250, 180, 150, 75, 38, 18, and 8 μm. The mass of material retained on each individual sieve was recorded, and the corresponding percentage by mass was calculated [40].

A homogeneous tailing fraction was prepared by screening the sample through a 150 μm sieve, after which the pH was determined by mixing this fraction with deionized water at a 1:5 ratio. To measure the total concentrations of Pb and Zn, the tailings waste underwent acid digestion using a mixture of hydrochloric acid, nitric acid, hydrofluoric acid, and perchloric acid, followed by analysis via analytikjena Plasma Quant 9000 Inductively Coupled Plasma Optical Emission Spectrometer (ICP-OES, Jena, Germany). The chemical fractionation of Pb and Zn within the tailings waste was characterized utilizing a sequential extraction technique, specifically the modified European Community Bureau of Reference (BCR) method [41,42].

#### 2.2.3. Basic Characterization Analysis of Granite Sawdust

The mineral composition of the granite sawdust was determined by XRD. The elemental oxide analysis was conducted using a PANalytical Axios X-ray fluorescence spectrometer (XRF, Almelo, The Netherlands). Furthermore, the microstructural features of the granite sawdust were examined using a TESCAN MIRALMS field-emission scanning electron microscope (SEM, Brno, Czech Republic). Elemental distribution mapping of Si, Al, C, and O across the observed interfaces was performed via Oxford energy-dispersive X-ray spectroscopy (EDS) coupled to the SEM system.

#### 2.2.4. Preliminary Synthesis of Granite Sawdust-Based Stabilizer

The granite sawdust-based stabilizer was synthesized primarily via the alkali fusion–hydrothermal method. Initially, measured quantities of granite sawdust and fly ash were separately immersed in 5% HCl solutions. The mixtures were magnetically stirred in a water bath maintained at 85 °C for 2~2.5 h. The acid-treated granite sawdust and fly ash were then isolated by negative pressure filtration, followed by thorough rinsing with deionized water until the pH was adjusted to neutrality.

**Figure 2:**
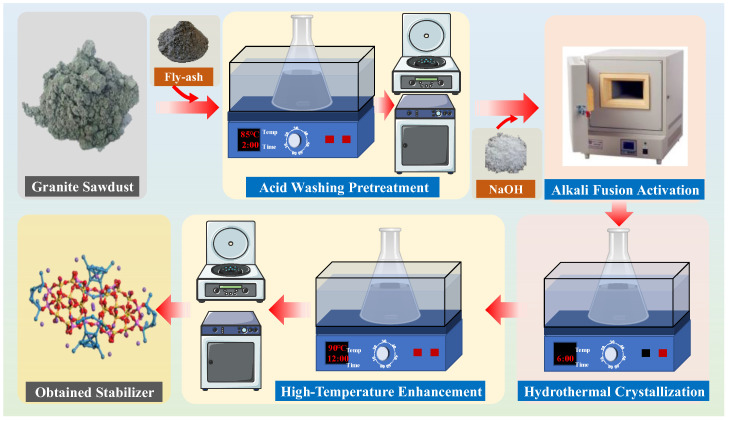
Preliminary synthesis procedure of stabilizer from granite sawdust.

Next, the dried acid-washed granite sawdust and a 2:1 mixture of sawdust and fly ash were separately blended with NaOH at a mass ratio of 1.2:1, followed by calcination in a muffle furnace at 750 °C for 4 h. After cooling to room temperature, the products were placed into 200 mL of deionized water and further reacted for 6 h under magnetic stirring. Subsequently, the solution temperature was raised to 90 °C, and the reaction continued for 12 h. The solid product was then washed, centrifuged, dried, and obtained two types of pH-neutral stabilizers (designated as GAS and GFA, respectively) (Figure 2). Finally, the stabilizers were mixed with the tailings waste samples at a ratio of 1:2, and the leaching amounts of Pb and Zn were determined through leaching experiments to compare their stabilizing effects on Pb and Zn elements in tailings waste.

#### 2.2.5. Modification and Optimization of Synthetic Products

**Figure 3:**
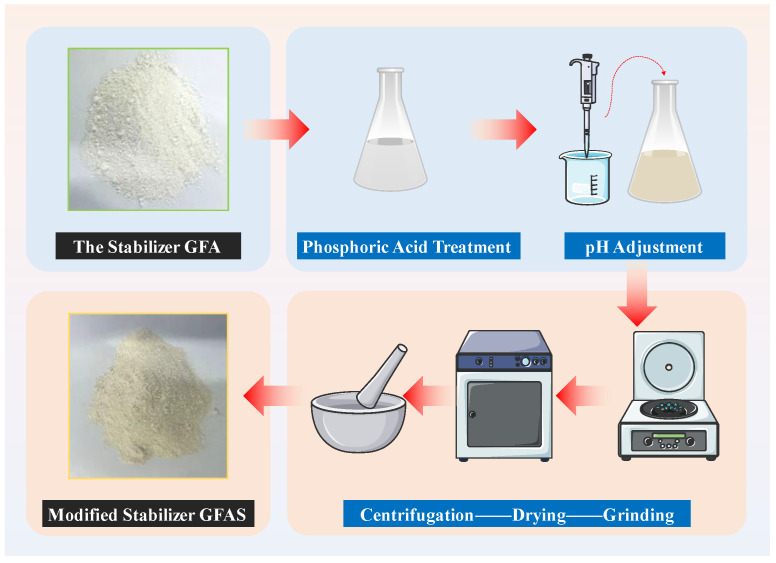
Modification Process of GFAS.

The synthesized stabilizer GFA was immersed in aqueous H_3_PO_4_ solutions at varying mass ratios and allowed to stand for 24 h with intermittent stirring. Following this treatment, the solids were recovered and repeatedly washed via centrifugation until the supernatant reached neutral pH. The pH was subsequently adjusted to 10~12 using 1 mol/L NaOH solution. The resulting phosphoric acid-modified granite sawdust-based stabilizer (designated GFAS) was obtained through sequential centrifugation, oven-drying, and pulverization, as schematically illustrated in Figure 3.

The stabilization efficacy of GFAS for lead (Pb) and zinc (Zn) in tailings waste was quantitatively assessed through the leaching experiment. To evaluate the temporal stability, varying masses of GFAS were blended with tailings waste and immersed in the leaching solution. Leachate concentrations of Pb and Zn were measured after 24 h and 30 days of contact time to determine the long-term immobilization performance.

#### 2.2.6. Phase and Structural Testing of GFAS

The mineral composition and elemental oxide composition of the GFAS were analyzed using XRD and XRF spectroscopy, respectively. Microstructural features were examined by SEM coupled with EDS, with elemental distribution mapping performed for Si, Al, O, Na, Fe, and P across selected interfacial regions. Characteristic functional groups of GFAS and granite sawdust were identified through Nicolet iS 50 Fourier-transform infrared (FT-IR, Madison, WI, USA) spectroscopy.

#### 2.2.7. Cation Exchange Capacity and Specific Surface Area Testing of GFAS

The cation exchange capacity (CEC) of stabilizer GFAS was determined using the hexaamminecobalt(III) chloride method [43]. Specifically, 3.5 g of dried granite sawdust and GFAS were separately placed into 100 mL centrifuge tubes, each containing 50.0 mL of 1.66 cmol/L [Co(NH_3_)_6_]Cl_3_ solution. The mixtures were agitated on a mechanical shaker for 60 min, followed by centrifugation to collect the supernatant for subsequent analysis. A calibration curve was established by measuring the absorbance of [Co(NH_3_)_6_]Cl_3_ standard solutions at concentrations of 0.000, 0.166, 0.498, 0.830, 1.160, and 1.490 cmol/L using Yipu V-T3 visible-light spectrophotometry (Yipu, China). Absorbance values (y-axis) were plotted against corresponding concentrations (x-axis) to generate the standard curve.

Using deionized water as the blank, the absorbance of the granite sawdust and GFAS supernatant samples was measured. The CEC values were then calculated according to Equation (1):

(1) $$\mathrm{CEC}\;=\;\frac{\left(A_{0}-A\;\right)\times V\;\times 3}{b\;\times m\;\times {\;W}_{dm}}$$

where *A*_0_ is the absorbance of the blank sample; *A* is the absorbance of the test sample; *V* is the volume of extractant solution, mL; 3 is the charge number of [Co(NH_3_)_6_]^3+^; *b* is the slope of the calibration curve; *m* is the sample mass, g; *W*_dm_ is the dry matter content of the sample, %.

The specific surface area of GFAS samples was determined via N_2_ adsorption–desorption analysis. Granite sawdust and GFAS specimens were first degassed under vacuum at 100 °C for 8 h. Subsequent N_2_ physisorption measurements were conducted at 77 K (−195.8 °C) using liquid N_2_ as the coolant in a Micromeritics APS 2460 four-station automated surface area analyzer (Atlanta, GA, USA). Adsorption–desorption isotherms obtained from the instrument were analyzed by the Brunauer–Emmett–Teller (BET) method to determine the total specific surface area.

#### 2.2.8. Adsorption Isotherms and Adsorption Kinetics of GFAS

To investigate the adsorption capabilities of GFAS, heavy metal stock solutions were prepared using analytical grade Pb(NO_3_)_2_·3H_2_O and Zn(NO_3_)_2_·6H_2_O. A concentration gradient was established for the adsorption isotherm experiments, with initial Pb^2+^ and Zn^2+^ concentrations set at 20, 40, 60, 80, 100, 150, and 200 mg/L, respectively. For each test, a fixed dosage of 0.05 g of GFAS was introduced into 50 mL of the ionic solution. These mixtures were subsequently agitated in an oscillator for 24 h to ensure equilibrium was reached. Following oscillation, the residual concentrations of Pb and Zn were quantified using Inductively Coupled Plasma Optical Emission Spectroscopy (ICP-OES).

For the adsorption kinetic analysis, the experimental conditions involved adding 0.05 g of GFAS to 100 mg/L solutions of Pb^2+^ and Zn^2+^. The samples were subjected to continuous oscillation and aliquots were withdrawn for ICP-OES analysis at specific time intervals: 1, 5, 10, 20, 30, 60, 90, and 240 min. The equilibrium adsorption capacity, denoted as *Q_e_*, was calculated based on Equation (2) [44,45]:

(2) $$Q_{e/t}=\frac{V(C_{0}-C_{e/t})10^{-3}}{W}$$

where *C*_0_ is the initial liquid concentration; *C*_*e*/*t*_ is the equilibrium liquid concentration or liquid concentration at any time, mg/L; *V* is the volume of ionic solution, here 50 mL; *W* is the amount of GFAS, here 0.05 g.

## 3. Results and Discussion

### 3.1. Physicochemical Characterization of Tailings Waste

**Figure 4:**
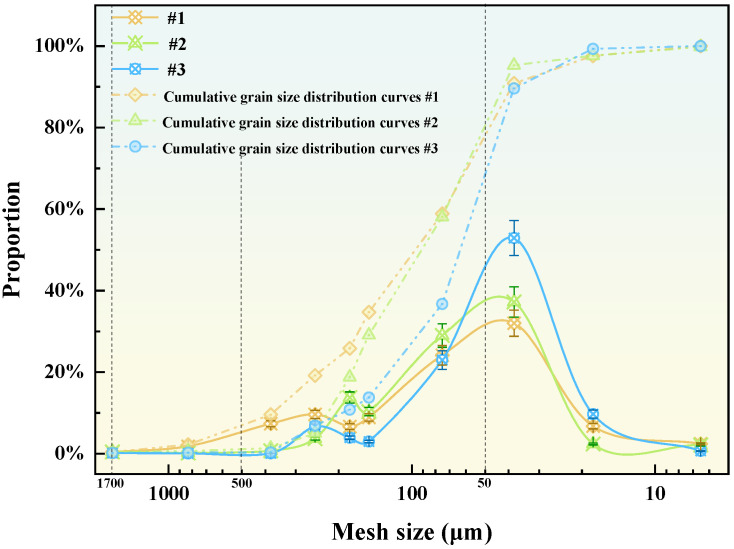
Particle size distribution of tailings waste.

The mineralogical composition of the tailings waste obtained from the Yunnan Shenxiandong lead-zinc pond is presented in Table 1. The analysis reveals that these carbonate-rich tailings waste is predominantly comprising siderite (FeCO_3_), quartz, and calcite. Furthermore, the particle size distribution, illustrated in Figure 4, demonstrates that most of the material falls within the 37~74 μm range. This fine fraction accounts for approximately 22% to 53% of the total mass.

Such fine-grained characteristics typically correspond to a larger specific surface area, thereby increasing the potential for heavy metal mobilization and release into the surrounding environment over prolonged periods. The tailings waste was determined to be acidic, with an average pH of 5.35 in aqueous solution. Due to their carbonate nature, the tailings waste is highly prone to acid-rock neutralization upon exposure to air and water, a process that further accelerates the leaching of heavy metal ions.

**Figure 5:**
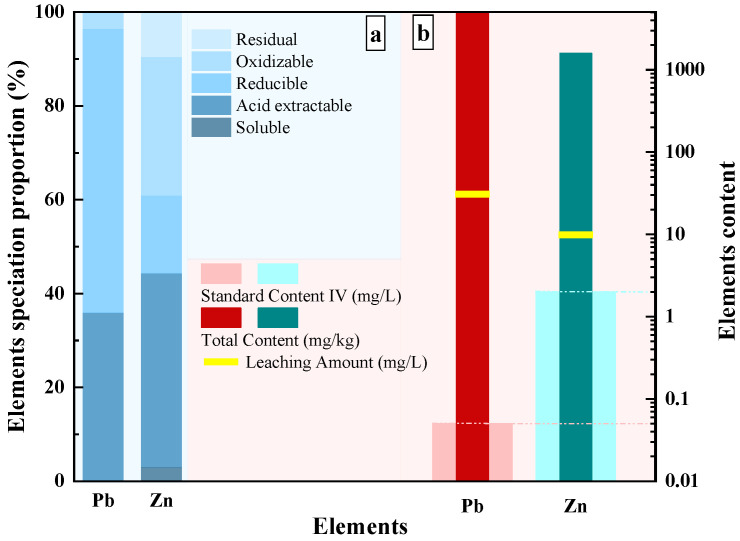
The proportion of lead and zinc in different forms in tailings. (a) The proportion of Pb and Zn speciation distribution; (b) Total content, leaching amount, and standard emissions of Pb and Zn in heavy metal tailings.

The speciation distribution of Pb and Zn in tailings waste is shown in Figure 5a. Among them, the combined proportion of soluble and acid-extractable Pb accounts for 35.9%, and Zn for 44.3%, exhibiting a strong migration behavior. This also indicates that the tailings waste can release significant amounts of Pb and Zn in acidic environments, which poses a significant environmental risk [46,47].

Figure 5b summarizes the total heavy metal content, leaching concentrations, and relevant regulatory standards for Pb and Zn. The acid-leaching results are particularly concerning; the concentration of Pb in the leachate exceeded the standard limit by a factor of 623. Similarly, the leaching amount of Zn was found to be 4.9 times higher than the permissible limit. These comparisons are based on the Class IV standards outlined in the Chinese National Standard for Environmental Quality Standards for Surface Water (GB/T 3838-2002), which serves as the benchmark for identifying pollution levels and setting limits for solid waste [48,49].

### 3.2. Characterization of Granite Sawdust Properties

**Figure 6:**
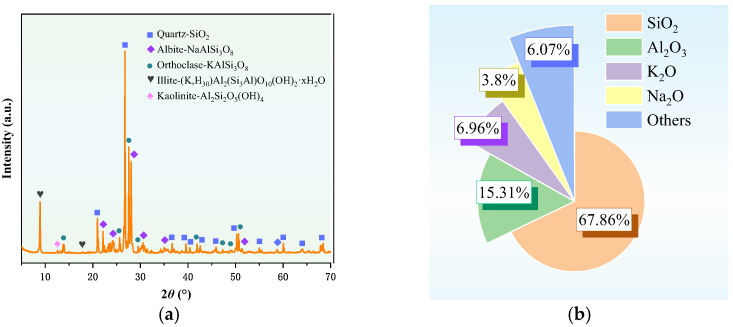
Mineral composition (XRD) and elemental oxide distribution (XRF) of the granite sawdust. (**a**) XRD pattern; (**b**) XRF results.

Figure 6a presents the XRD pattern of granite sawdust, while Figure 6b shows the relative abundance of elemental oxides in the same material. The results demonstrate that granite sawdust is primarily composed of quartz, albite, potassium feldspar, illite, and kaolinite. Further compositional analysis indicates that SiO_2_ constitutes the primary oxide component (67.86%), with Al_2_O_3_ representing the secondary component (15.31%). These results indicate that granite sawdust contains significant amounts of silicon (Si) and aluminum (Al), which are essential for the synthesis of zeolite-based stabilizers via the alkali fusion–hydrothermal method.

**Figure 7:**
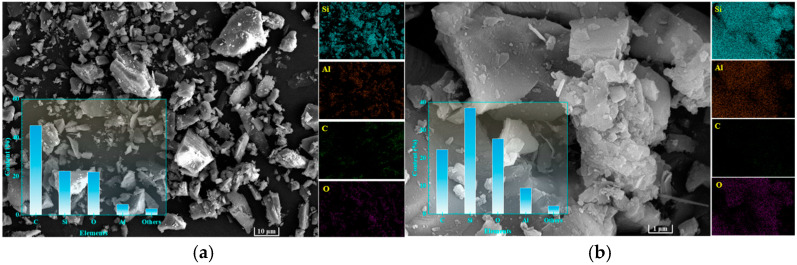
SEM and EDS images of granite sawdust. (**a**) ×2200 magnification (**b**) ×22,000 magnification.

Figure 7 presents SEM micrographs of granite sawdust at different magnifications. As depicted in Figure 7a, the majority of the particles are observed to be greater than 10 μm in size, a finding that is in agreement with the data obtained through sieving analysis. At higher magnification (×20,000; Figure 7b), particles exhibit smooth surfaces lacking effective adsorption sites for Pb and Zn. EDS mapping (Figure 7) reveals Si, Al, C, and O as the primary constituents. These elements are derived principally from quartz, albite, and potassium feldspar minerals. Granite sawdust exhibits negligible adsorption capacity for heavy metals, primarily due to its low specific surface area and lack of surface features favorable for adsorption. Nevertheless, the high contents of Si, Al, and O provide a favorable elemental basis for the synthesis of zeolite-based stabilizers through appropriate processing methods.

### 3.3. Analysis of the Preliminary Synthesis of Granite Sawdust-Based Stabilizer

**Figure 8:**
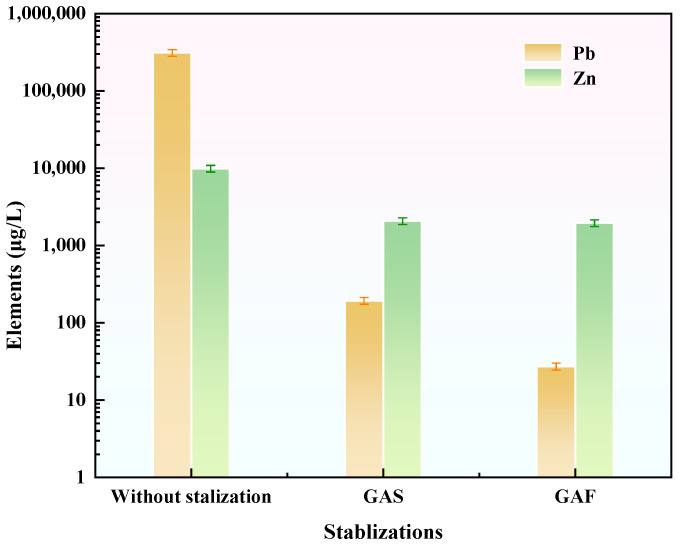
Stabilization efficacy of different stabilizers on Pb and Zn in tailings waste.

As demonstrated in Figure 8, the stabilizer GAS exhibited significant immobilization of Pb and Zn in tailings waste. In comparison with tailings waste lacking a stabilizer, the incorporation of GAS resulted in a 99.9% reduction in Pb leaching and a 79.1% reduction in Zn leaching, thereby substantiating its efficacy in stabilizing these heavy metals.

It is noteworthy that the stabilizer GFA demonstrated a superior adsorption efficacy in comparison to GAS. Specifically, compared to GAS, GFA achieved an additional 85.9% reduction in Pb leaching and a 5.5% reduction in Zn leaching. Therefore, GFA was selected for further investigation as the most suitable stabilizer for the immobilization of Pb and Zn in tailings waste.

### 3.4. Modification of Synthesized Granite Sawdust-Based Stabilizer

**Figure 9:**
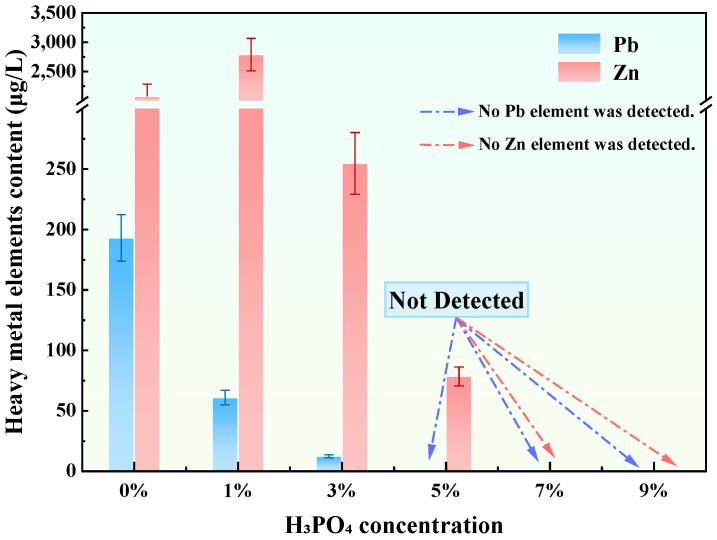
Stabilization efficacy of GFAS modified with different H_3_PO_4_ concentrations on Pb and Zn release from tailings waste.

Leaching tests conducted on tailings waste treated with GFAS, the H_3_PO_4_-modified stabilizer derived from GFA, are presented in Figure 9. The results indicate a significant enhancement in the immobilization capacity for Pb and Zn following H_3_PO_4_ treatment. At 3wt% H_3_PO_4_, leachate concentrations decreased to 12.41 μg/L (Pb) and 254.7 μg/L (Zn), representing 54.6% and 87.0% reductions relative to GFA-treated tailings waste, respectively. The stabilization effect improves significantly with increasing H_3_PO_4_ dosage. At a concentration of 5 wt%, Pb^2+^ was no longer detected in the leachate. When the dosage was increased to 7 wt%, both Pb^2+^ and Zn^2+^ concentrations dropped below the detection limits. To further verify this trend, an additional test with 9 wt% H_3_PO_4_ was conducted, which yielded identical results (non-detectable leaching). These results indicate that 7 wt% represents the threshold concentration required to achieve complete immobilization of heavy metals in tailings waste. Consequently, considering the balance between stabilization performance and cost-effectiveness, 7 wt% H_3_PO_4_ was selected as the most effective dosage for subsequent analysis.

**Figure 10:**
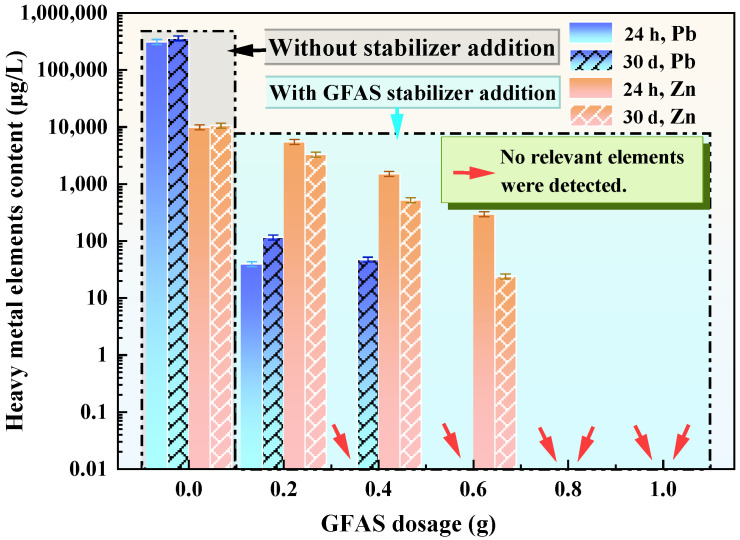
Stabilization efficacy of different GFAS dosages on Pb and Zn in tailings waste.

Temporal stability assessments comparing 24 h and 30-day leaching experiments (Figure 10) revealed significantly reduced heavy metal leaching from tailings waste treated with GFAS. At a 0.2 g dosage, Pb leaching decreased from 39,340 μg/L to 39.6 μg/L (99.9% reduction), while Zn leaching decreased from 9935 μg/L to 5495 μg/L (44.7% reduction) after 24 h treatment. Leachate concentrations of both elements exhibited inverse correlation with GFAS dosage. At dosages ≥0.8 g, Pb and Zn were below detection limits in leachates following both 24 h and 30-day exposures. Notably, identical GFAS dosages yielded further decreased metal concentrations after 30 days compared to the 24 h results. These results clearly demonstrate the excellent long-term stabilization performance of the synthesized GFAS for Pb and Zn immobilization in tailings waste.

### 3.5. Phase and Structural Analysis of GFAS

**Figure 11:**
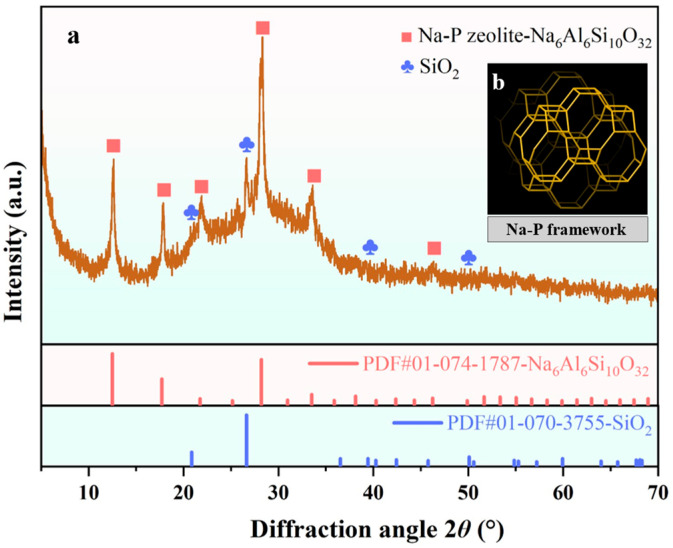
XRD pattern of GFAS and Na-P framework. (**a**) XRD pattern of GFAS. (**b**) Na-P framework.

Figure 11a presents the XRD pattern of GFAS. It primarily consists of synthesized Na_6_Al_6_Si_10_O_32_ and a minor fraction of incompletely reacted SiO_2_ particles. Based on a comparison with the zeolite structure database of the International Zeolite Association (IZA) and references, GFAS was confirmed to be a Na-P type zeolite (Na_6_Al_6_Si_10_O_32_) through the synthesis sequence from granite sawdust, with the corresponding structure illustrated in Figure 11b [50,51].

**Figure 12:**
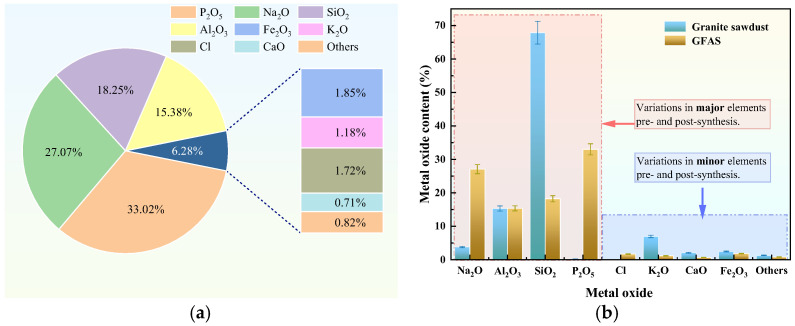
XRF analysis of GFAS and granite sawdust. (**a**) XRF result of GFAS. (**b**) Comparative XRF analysis: GFAS vs. sawdust.

Figure 12a shows the elemental oxide composition of GFAS. The predominant oxides are P_2_O_5_ (33.02%), Na_2_O (27.07%), SiO_2_ (18.25%), and Al_2_O_3_ (15.38%). The elevated P_2_O_5_ content primarily originated from the H_3_PO_4_ modification treatment, where (PO_4_)^3−^ anions incorporated into the zeolitic structure and combined with oxygen elements during synthesis. This molecular reorganization resulted in P_2_O_5_ detection by XRF, confirming successful structural integration of (PO_4_)^3−^ groups within the GFAS matrix. Meanwhile, SiO_2_ and Al_2_O_3_ were predominantly derived from the synthesized zeolite (Na_6_Al_6_Si_10_O_32_). The presence of chloride ions in the synthesized GFAS, as identified in the elemental analysis, is attributed to the acid washing pretreatment stage, where hydrochloric acid (HCl) was employed to activate the raw materials (granite sawdust and fly ash). Although a NaOH solution was subsequently introduced to adjust the pH and provide an alkaline environment for zeolite crystallization, residual chloride species from the initial HCl treatment were partially retained within the silicate framework or on the surface of the particles.

A comparative analysis of the XRF results between the sawdust and GFAS is presented in Figure 12b. During the alkali fusion–hydrothermal process, the content of SiO_2_ was significantly reduced. This reduction primarily occurred because, beyond the portion of Si and Al elements combining to form zeolite (Na_6_Al_6_Si_10_O_32_), the excess SiO_2_ reacted with NaOH at elevated temperatures to generate water-soluble sodium silicate (Na_2_SiO_3_). This compound was subsequently removed during the subsequent hydrothermal treatment via sequential washing and filtration processes. Consequently, the final GFAS product retained silicon in a stoichiometric ratio commensurate with aluminum. In addition, a significant increase in Na2O content was observed subsequent to the synthesis reaction. This is primarily attributed to the introduction of substantial NaOH during the process, which served as the sodium source, thereby leading to the elevated Na_2_O levels.

**Figure 13:**
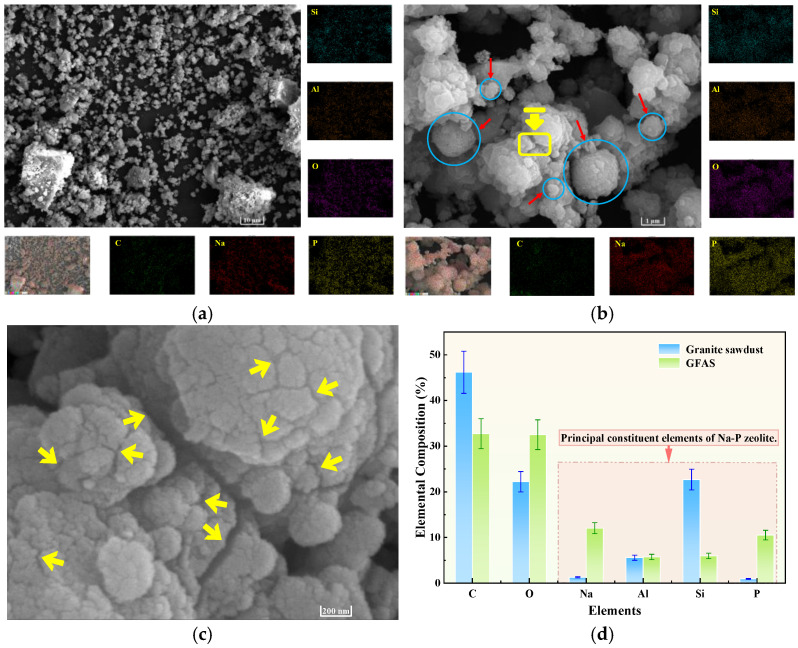
SEM and EDS images of GFAS. (**a**) ×2200 magnification. (**b**) ×22,000 magnification (The red arrows and blue circles are used to indicate the morphology of the GFAS particles, while the yellow rectangles mark the regions selected for further magnified observation in (**c**)). (**c**) ×110,000 magnification. (**d**) EDS Comparison of the sawdust and GFAS (×2200).

The microstructure of GFAS was examined using SEM, coupled with EDS for elemental mapping analysis. The distribution of key elements, including Si, Al, C, O, Na, and P, across the scanned interface is presented in Figure 13.

Figure 13a presents an SEM micrograph of GFAS at ×2200 magnification. Compared to the granite sawdust at an identical scale (Figure 7a), the majority of GFAS particles are smaller than 10 μm and exhibit a non-smooth surface morphology. Upon higher-magnification examination (×22,000, Figure 13b), GFAS particles demonstrate a more uniform size distribution and a rougher surface topography relative to the sawdust (Figure 7b). The particles primarily display irregular spherical morphologies (denoted by the blue line in Figure 13b), which are identified as agglomerates of Na-P zeolite particles. This observation is consistent with findings reported by Pameli et al. and others [52,53,54].

Further magnification of the yellow rectangular region in Figure 13b to ×110,000 (Figure 13c) reveals distinct porous structures on the GFAS particle surface (indicated by yellow arrows). These pores contribute significantly to the increased specific surface area and porosity of the material.

EDS elemental analysis of GFAS (Figure 13) revealed Si, Al, O, and P as the predominant uniformly distributed elements. The significantly increased and homogeneous distribution of P confirms the successful incorporation of phosphorus through phosphoric acid modification. A comparative analysis of the elemental composition between granite sawdust and GFAS at identical SEM magnification (×2200) is presented in Figure 13d.

The Si content decreased markedly by 73.7% after synthesis, whereas Al remained largely unchanged. This disparity primarily occurs because, under fixed synthesis conditions, Si and Al react in a fixed stoichiometric ratio to form zeolite. The excess Si subsequently reacts with NaOH to form water-soluble sodium silicate (Na_2_SiO_3_), which is removed during the subsequent hydrothermal treatment via washing and filtration steps. Conversely, the contents of Na and P increased significantly. This is attributed to the extensive use of NaOH, which served as both the alkaline activator and pH regulator, providing a substantial sodium source, and the introduction of phosphoric acid, which contributed to the increased P content. These EDS findings are consistent with the XRF results (Figure 12).

**Figure 14:**
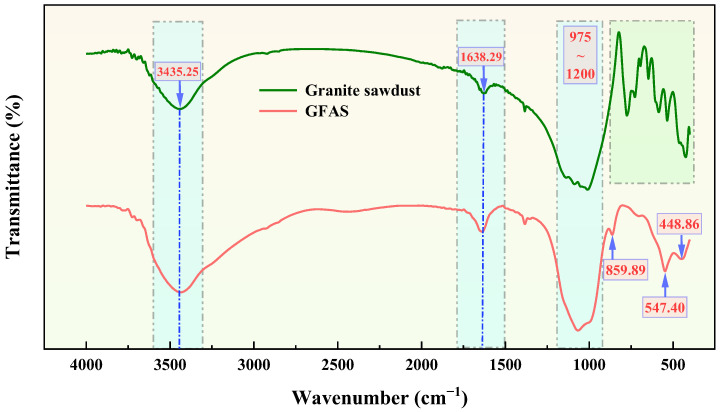
FT-IR spectra of granite sawdust and GFAS (The green shaded area represents the characteristic fingerprint region of granite minerals, while the blue shaded area indicates the main spectral regions showing changes before and after the synthesis of the sawdust).

The FT-IR spectrum of GFAS is presented in Figure 14. The absorption bands in the 400~800 cm^−1^ range (light green shaded area) are primarily attributed to characteristic vibrations of granite minerals [55]. The peaks observed at 1638.29 and 3435.25 cm^−1^ correspond to the bending vibration and asymmetric stretching vibration of -OH groups, respectively. These hydroxyl groups likely originate from structurally bound water in both the original minerals and the synthesized stabilizer. The broad absorption band between 975 and 1200 cm^−1^ is assigned to the asymmetric stretching vibrations of T-O bonds (T = Si or Al) in both granite sawdust and GFAS. The peak at 448.86 cm^−1^ is attributed to the bending vibrations of these T-O bonds. The observed spectral shifts between the precursor and GFAS are primarily due to alterations in the coordination environment and bonding ratios among Si, Al, and O atoms after synthesis. Notably, the FT-IR spectrum of GFAS exhibits characteristic peaks of Na-P zeolite. The peak at 547.40 cm^−1^ is indicative of the double-ring topological structure specific to Na-P zeolite. Additionally, peaks associated with HOPO_3_^2−^ groups are observed: a bending vibration at 859.89 cm^−1^ and asymmetric stretching vibrations within the 1000~1200 cm^−1^ range [53,56,57,58]. These features confirm the successful incorporation of phosphate ions into the stabilizer matrix, which is consistent with the prior findings from SEM, EDS, and XRF analyses.

### 3.6. Cation Exchange Capacity and Specific Surface Area Analysis of GFAS

**Figure 15:**
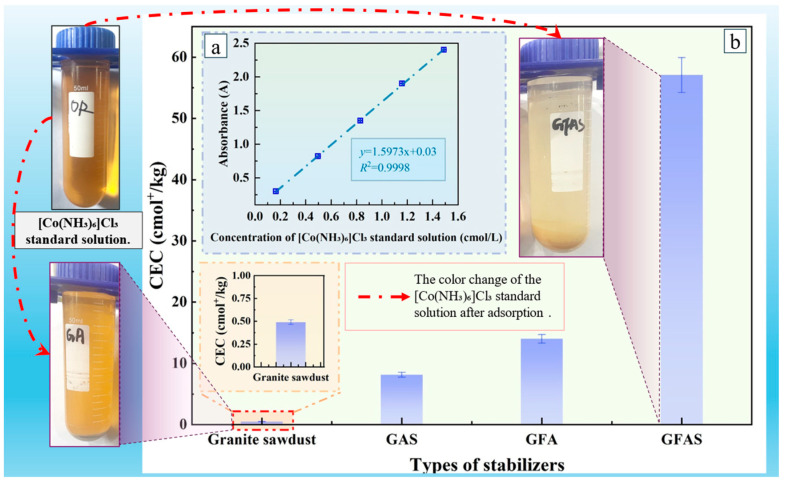
Cation exchange capacity (CEC) values of different stabilizers. (**a**) Fitting curve of [Co(NH_3_)_6_]Cl_3_ standard solution. (**b**) Cation exchange capacity (CEC) values of different stabilizers.

Figure 15 presents the cation exchange capacity (CEC) values of different stabilizers, calculated based on Equation (1) and the adsorption curve of the [Co(NH_3_)_6_]Cl_3_ standard solution. The Figure also illustrates the visual color change in the solution before and after the adsorption process. The CEC of the granite sawdust was determined to be only 0.49 cmol^+^/kg, confirming its poor inherent cation exchange capability. This result is consistent with the negligible color change observed in Figure 15. Conversely, the synthesized GFAS exhibited a remarkably high CEC of 57.08 cmol^+^/kg, signifying a 116-fold augmentation compared to the initial sawdust sample. This exceptionally enhanced cation exchange capacity directly corresponds to the near-complete decolorization of the [Co(NH_3_)_6_]Cl_3_ solution. Figure 15 illustrates the color changes in the [Co(NH_3_)_6_]Cl_3_ standard solution after 24 h of adsorption by granite sawdust and GFAS. This provides visual evidence of GFAS’s superior adsorption capacity.

Meanwhile, the CEC value of GFAS increased by 4- to 7-fold compared to GAS and GFA. These results demonstrate that the synergistic effect of phosphoric acid modification and fly ash incorporation significantly enhances the cation exchange capacity and heavy metal adsorption capability of the synthesized stabilizers.

N_2_ adsorption–desorption experiments were conducted on GFAS and granite sawdust to characterize the specific surface area and primary pore size distribution of GFAS. The results are summarized in Table 2.

The specific surface area of the sawdust was only 3.68 m^2^/g, significantly lower than that of typical stabilizers or adsorbent materials. This exceptionally low surface area resulted in extensively negative N_2_ adsorption values during measurement, indicating that the granite sawdust is predominantly non-porous or contains minimal micropores (0~2 nm) and mesopores (2~50 nm) [59]. Therefore, only the BET-specific surface area data of the sawdust could be obtained from the experimental results.

In contrast, the measured BET surface area of GFAS was 35.00 m^2^/g, approximately 10 times greater than that of the sawdust. GFAS exhibited a micropore area of 6.08 m^2^/g and a BJH adsorption average pore diameter (4 V/A) of 7.28 nm. These results demonstrate that GFAS is a micro-mesoporous material with substantially enhanced specific surface area, which contributes directly to its improved heavy metal adsorption capacity.

**Figure 16:**
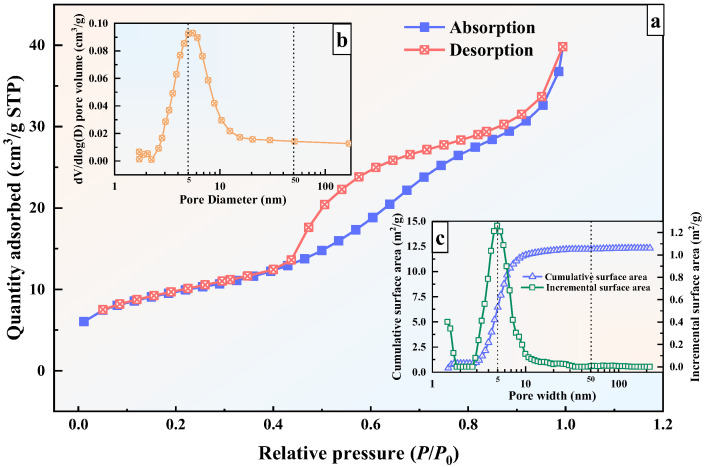
Results of N_2_ adsorption-desorption experiments in GFAS. (**a**) N_2_ adsorption–desorption isotherm of GFAS. (**b**) Pore size distribution of GFAS. (**c**) Surface ares distribution of GFAS.

The N_2_ adsorption–desorption isotherm of GFAS is presented in Figure 16a. The isotherm can be classified as Type IV with an H4-type hysteresis loop, which is typically associated with solids containing narrow slit-like pores, such as aggregated zeolite crystals, certain mesoporous zeolites, and micro-mesoporous materials [58,60]. This observation is consistent with the SEM results, which revealed the presence of micro-nano scale crack-like pores on the surface of GFAS particles.

The pore size distribution and specific surface area profile of GFAS are also presented in Figure 16b,c. As shown in Figure 16b, the predominant pore size evolution occurs within the 3~10 nm range, which substantially contributes to the enhanced specific surface area of GFAS. Figure 16c further demonstrates that the incremental increase in specific surface area primarily originates from pores within this same 3~10 nm range. The cumulative surface area within the 1.48~200 nm range measures 12.32 m^2^/g, with pores in the 1~10 nm range contributing 11.64 m^2^/g, accounting for 94.6% of the total specific surface area. This indicates that the specific surface area of GFAS is predominantly derived from pores measuring 3~10 nm, which is consistent with the previously reported BJH adsorption average pore diameter (4 V/A) of 7.28 nm (Table 2). These results collectively confirm that GFAS is a micro-mesoporous material.

### 3.7. Analysis of Adsorption Isotherms and Adsorption Kinetics of GFAS

To evaluate the adsorption capacity and underlying mechanism of GFAS, the experimental data were analyzed using two classic theoretical models: Langmuir and Freundlich. These models are mathematically represented in Equations (3) and (4), respectively [44]:

(3) $$Q_{e}=\frac{Q_{m}K_{l}C_{e}}{1+K_{l}C_{e}}$$

(4) $$Q_{e}=K_{f}{C_{e}}^{\frac{1}{n}}$$

To facilitate the calculation of characteristic parameters and assess the goodness of fit, these non-linear equations were converted into their linear forms. The linearized Langmuir and Freundlich expressions are presented in Equations (5) and (6):

(5) $$\frac{C_{e}}{Q_{e}}=\frac{C_{e}}{Q_{m}}+\frac{1}{{K_{l}Q}_{m}}$$

(6) $$logQ_{e}=\left(\frac{1}{n}\right)logC_{e}+logK_{f}$$

where *Q_m_* is the maximum adsorption capacity, mg/L; K_l_ is the Langmuir equilibrium constant; K_f_ is the Freundlich equilibrium constant; n is the Freundlich adsorption strength constant.

Regarding the adsorption rate, the solute uptake behavior of the stabilizing agents was examined using kinetic modeling. The specific equations for the Lagergren pseudo-first-order and pseudo-second-order models are detailed in Equations (7) and (8) [45]:

(7) $$\log\left(Q_{e}-Q_{t}\right)=logQ_{e}-\frac{K_{1}}{2.303}t$$

(8) $$\frac{t}{Q_{t}}=\frac{1}{K_{2}{Q_{e}}^{2}}+\frac{t}{Q_{e}}$$

where K_1_ is the pseudo-first-order rate constant; K_2_ is the pseudo-second-order rate constant; *t* is the adsorption time, min.

**Figure 17:**
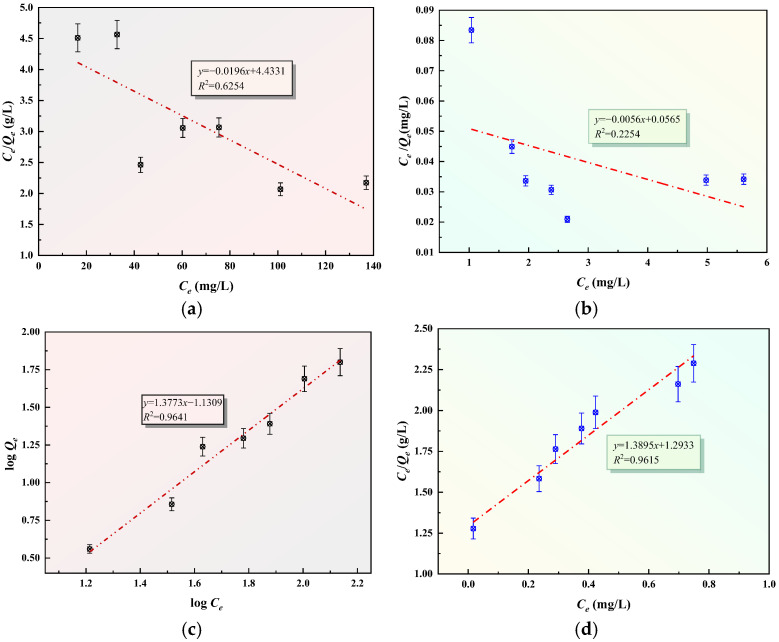

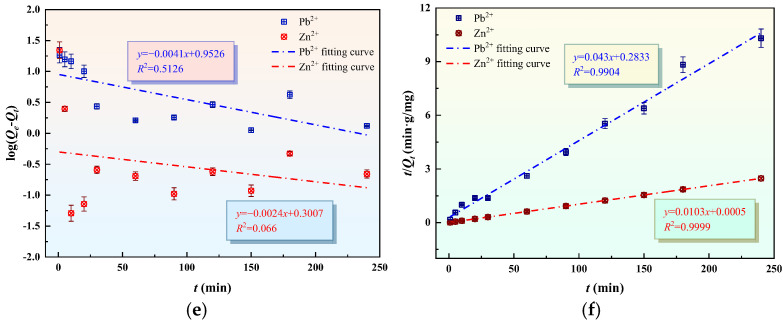
Adsorption isotherms and adsorption kinetics of GFAS to Pb^2+^ and Zn^2+^. (**a**) Langmuir adsorption for Pb^2+^. (**b**) Langmuir adsorption for Zn^2+^. (**c**) Freundlich adsorption for Pb^2+^ (**d**) Freundlich adsorption for Zn^2+^. (**e**) The pseudo-first-order kinetic. (**f**) The pseudo-second-order kinetic.

Linear regression analysis was performed to validate the isotherm models. As shown in Figure 17a–d, the plots of *C_e_*/*Q_e_* versus *C_e_* (Langmuir) and log*Q_e_* versus log*C_e_* (Freundlich) were generated. A comparison of the correlation coefficients *R*^2^ reveals that the adsorption behavior of Pb^2+^ and Zn^2+^ on GFAS aligns more closely with the Freundlich isothermal model than the Langmuir model. This suggests that the adsorption occurs on a heterogeneous surface with multilayer coverage. The kinetic data were analyzed by plotting log(*Q_e_*−*Q_t_*) and *t*/*Q_t_* against time, as illustrated in Figure 17c–f. The statistical results demonstrate that the pseudo-second-order kinetic model provides an exceptional fit for both metal ions, with *R*^2^ values exceeding 0.99. This high correlation implies that the removal process is not limited by a single step; rather, it reflects a complex mechanism that likely incorporates surface chemisorption, film diffusion, and intra-particle diffusion steps simultaneously.

### 3.8. Mechanism of Lead and Zinc Stabilization by GFAS

The stabilization mechanism of Pb and Zn in tailings waste by the synthesized zeolite-based stabilizer GFAS is analyzed as follows. The efficient immobilization of Pb and Zn by GFAS is governed by the synergistic effects of multiple simultaneous mechanisms.

**Figure 18:**
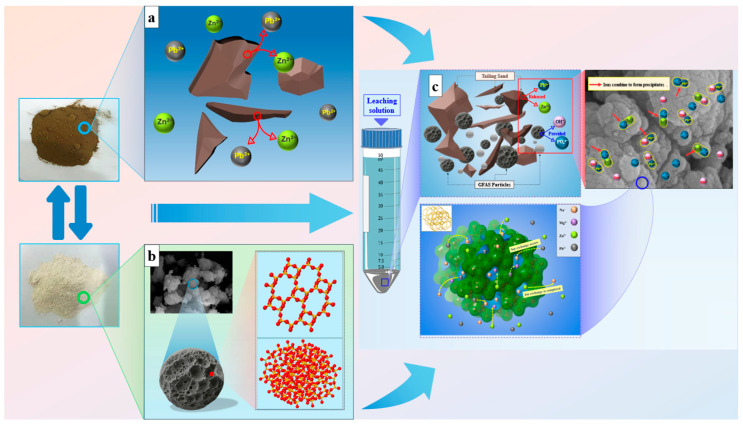
Mechanism diagram of GFAS for stabilizing Pb and Zn. (**a**) Release of heavy metals from tailings into solution. (**b**) Microstructure of GFAS. (**c**) Mechanism of Pb and Zn stabilization in tailings by GFAS.

Phase analysis identifies GFAS as a Na-P type zeolite. The crystal structure of this zeolite type (Figure 18b) contains distinctive cage-like configurations. These cage-like structures contribute to a complex porous network and a high specific surface area, consistent with the micro-mesoporous material classification determined by N_2_ physisorption experiments. GFAS exhibits a complex surface morphology with abundant porous structures, as clearly observed in SEM images (Figure 13). The specific surface area of 35.00 m^2^/g and average pore diameter of 7.28 nm, measured via N_2_ adsorption, confirm the unique crystalline framework and extensive porosity characteristic of zeolites. This structural configuration provides a substantially increased surface area for interaction with Pb^2+^ and Zn^2+^, thereby enhancing their immobilization efficiency. The initial adsorption is primarily governed by van der Waals interactions between the metal ions and the GFAS surface, resulting in rapid but relatively weak physical adsorption [61].

Furthermore, analytical results from XRF (Figure 12), EDS (Figure 13d), and FT-IR (Figure 14) confirm that H_3_PO_4_ treatment and pH adjustment with NaOH during synthesis introduced abundant hydroxyl (-OH) and phosphate (-HOPO_3_) groups on the GFAS surface (Figure 18c). These functional groups react with Pb^2+^ and Zn^2+^ through Equations (9)–(12), forming poorly soluble precipitates/complexes such as Pb(OH)_2_, Zn(OH)_2_, Zn_3_(PO_4_)_2_, and Pb_3_(PO_4_)_2_ stabilized on the zeolite surface. The low solubility of these compounds effectively reduces the mobility and bioavailability of the heavy metal ions.

Pb^2+^ + 2OH^−^ → Pb(OH)_2_↓ (9)

Zn^2+^ + 2OH^−^ → Zn(OH)_2_↓ (10)

2(PO_4_)^3−^ + 3Zn^2+^→ Zn_3_(PO_4_)_2_↓ (11)

2(PO_4_)^3−^ + 3Pb^2+^→ Pb_3_(PO_4_)_2_↓ (12)

The alkaline synthesis conditions (pH 11~12) imparted a strong negative surface charge to GFAS, enhancing electrostatic interactions with heavy metal cations. Simultaneously, the high OH^−^ concentration promoted metal hydroxide precipitation on the zeolite surface [61,62]. As reported by Painer et al., the synthesis of zeolites from aluminosilicate precursors is highly dependent on the dissolution-precipitation kinetics during alkali treatment. GFAS possesses a more active mesoporous structure. This high reactivity is essential for the rapid immobilization of Pb and Zn in tailings, as it provides a higher density of Na^+^ exchange sites and phosphate-active centers for chemical precipitation [63].

Furthermore, during the synthesis process, NaOH provides a substantial amount of Na^+^ ions, which become extensively and uniformly distributed both on the surface and within the internal structure of GFAS particles, specifically occupying channels and cages of the zeolitic framework. Due to the relatively weak binding force between Na^+^ ions and the zeolitic framework composed of [SiO_4_] and [AlO_4_] tetrahedra, these ions are readily exchanged when other cations, such as Pb^2+^ and Zn^2+^ released from the tailings waste into the solution, are present (Figure 18a) [64,65]. Concurrently, inherent Mg^2+^ and Ca^2+^ ions within GFAS also participate in this ion exchange process, further contributing to the immobilization of heavy metal cations like Pb^2+^ and Zn^2+^ (Figure 18c).

In summary, the adsorption of Pb^2+^ and Zn^2+^ by GFAS proceeds through multiple mechanisms, including physical adsorption facilitated by its unique zeolitic structure, high specific surface area, chemical adsorption via the formation of metal hydroxides and phosphate precipitates, and ion exchange between cations. This synergistic effect results in remarkable adsorption and stabilization efficacy for Pb^2+^ and Zn^2+^. The granite sawdust-based stabilizer (GFAS) demonstrates superior performance in immobilizing Pb and Zn in tailings. The higher cation exchange capacity (CEC) and specific surface area of GFAS enhance its efficiency, positioning it as a promising alternative to traditional stabilizers. Furthermore, when compared with traditional physical methods like soil stabilization and thermal treatment, and physicochemical methods such as chemical precipitation, GFAS offers a more sustainable and effective solution for long-term heavy metal immobilization [66].

Beyond environmental remediation, rather than being treated as a problematic waste for storage, granite sawdust should be recognized as a valuable secondary source of feldspar-quartz mineral flour and others. Here, by employing the alkali fusion–hydrothermal method, these mineral resources are successfully upcycled into high-value functional adsorbents (GFAS). This “waste-treats-waste” model not only addresses the ecological risks of heavy metal tailings but also promotes the sustainable circulation of industrial by-products. Such a strategy reduces the reliance on virgin materials and provides a commercially and environmentally viable pathway for the stone processing and mining industries to achieve closed-loop resource management. Based on this, the subsequent stage of the project will entail the development of a GFAS-cement mortar composite, the purpose of which will be to seal leakage points in tailings pond foundations. The objective of this approach is twofold: firstly, to enhance the impermeability of the solidified matrix; and secondly, to immobilize heavy metals present in the tailings waste, thus preventing their migration through cracks.

## 4. Conclusions

The main conclusions of this study are summarized as follows:

(1) The tailings waste particles present in the Shenxiandong Lead-Zinc tailings pond are distinguished by their diminutive size and proclivity for the release of lead (Pb) and zinc (Zn) into the surrounding environment. Experiments involving leaching demonstrate that the capacity of the subjects to release lead (Pb) and zinc (Zn) exceeds the standard limit by 623 times and 4.9 times, respectively. This indicates a significant potential for environmental risk.

(2) Granite sawdust has small particle sizes, mainly distributed in the range of 75~150 μm, which brings potential hazards of being easily blown away by wind and causing soil erosion. Its microscopic surface is smooth, lacking adsorption sites for Pb and Zn. It is mainly composed of quartz, albite, potassium feldspar, illite, and kaolinite, with silicon (Si), aluminum (Al), and oxygen (O) as the main constituent elements. These elements provide the elemental basis for synthesizing zeolite-based stabilizers from granite sawdust.

(3) By treating granite sawdust via the alkali fusion–hydrothermal method and modifying it with 7%H_3_PO_4_ solution, a stabilizer (GFAS) with significant stabilization efficiency for Pb and Zn in tailings waste was synthesized. When GFAS was added at a mass ratio of 0.8~1:2 (GFAS to tailings waste), no leaching of Pb or Zn was detected after 24 h and 30 days, confirming that GFAS exhibits remarkable and long-term stabilization performance.

(4) GFAS has smaller and more uniform microscopic particle sizes, with abundant crack characteristics on its surface. XRD, EDS, and FT-IR analyses reveal that GFAS is mainly composed of Na-P type zeolite (Na_6_Al_6_Si_10_O_32_). Its CEC value is 57.08 cmol^+^/kg (much higher than that of granite sawdust), specific surface area is 35.00 m^2^/g, and average pore size is 7.28 nm, classifying it as a zeolite-based micro-mesoporous material. Additionally, its surface contains hydroxyl and phosphate groups, which provide the structural and chemical basis for Pb and Zn adsorption.

(5) The adsorption of Pb and Zn by GFAS conforms to the Freundlich isotherm model (*R*^2^ > 0.94) and the pseudo-second-order kinetic model (*R*^2^ > 0.99). GFAS achieves efficient stabilization of Pb and Zn in tailings waste through synergistic mechanisms including physical adsorption (via van der Waals forces), chemical precipitation (e.g., forming phosphate precipitates), and ion exchange (e.g., with Na^+^). This makes it an effective material for remediating heavy metal pollution.

## Figures and Tables

**Table 1:** Mineral composition of tailings waste.

Composition Types	Sample 1#	Sample 2#	Sample 3#
Quartz (%)	41.77	24.02	6.18
Siderite (%)	21.25	34.61	40.48
Calcite (%)	10.01	18.90	24.29
Dolomite (%)	0	13.55	0
Olivine (%)	21.46	0	0
Illite (%)	3.72	0	0
Clinoptilolite (%)	0	8.92	0
Ferrous dolomite (%)	0	0	29.05
Wollastonite (%)	1.8	0	0

**Table 2:** Analysis of specific surface area of granite sawdust and GFAS.

Content	Granite Sawdust	GFAS
BET surface area	3.68 m^2^/g	35.00 m^2^/g
t-Plot micropore area	——	6.08 m^2^/g
BJH adsorption cumulative surface area of pores between 1.7 nm and 300.0 nm diameter	——	32.81 m^2^/g
BJH Adsorption average pore diameter (4 V/A)	——	7.28 nm

## Data Availability

The original contributions presented in this study are included in the article. Further inquiries can be directed to the corresponding authors.
